# Nature-based solutions for coastal protection in sheltered and exposed coastal waters: integrated monitoring program for baseline ecological structure and functioning assessment

**DOI:** 10.1007/s10661-024-12480-x

**Published:** 2024-02-28

**Authors:** Arnaud Boulenger, Pablo Lanza-Arroyo, Kobus Langedock, Alexia Semeraro, Gert Van Hoey

**Affiliations:** 1 Laboratory of Oceanology, MARE Centre, UR FOCUS, University of Liège, 11 allée du six août, 4000 Liège, Belgium; 2 Laboratorio de Biología Marina, Departamento de Zoologia, Universidad de Sevilla, E-41012 Seville, Spain; 3 Flanders Marine Institute (VLIZ), Jacobsenstraat 1, 8400 Ostend, Belgium; 4 Flanders Research Institute for Agriculture, Fisheries and Food (ILVO), Jacobsenstraat 1, 8400 Ostend, Belgium; 5 IMBRSea, Marine Biology Research Group, Ghent University, Krijgslaan 281/S8, 9000 Ghent, Belgium

**Keywords:** Biogenic reefs, *Mytilus edulis*, Benthic monitoring, Ecosystem functioning, Biotic indices

## Abstract

Nature-based solutions, such as shellfish reefs, can support natural coastal defence and be a potential solution for climate-resilient shorelines in the future. In the Belgian Part of the North Sea, the “Coastbusters” projects aim to develop nature-based coastal protection by favouring subtidal mussel bed establishment on the seafloor through typical longline aquaculture techniques. Mussel beds are dependent on environmental conditions, and both influence the physical and biogeochemical features in a soft-sediment environment. Therefore, a comprehensive ecological monitoring program is essential to assess the success of future mussel bed development and its influence on the surrounding ecosystem. For establishing a monitoring baseline of the two experimental areas, a combination of conventional benthic assessment methods (grab sampling and granulometry) and non-invasive techniques (sediment profile imaging and transect diving video surveys) were utilised. Although mussel reefs did not yet develop by the time of this study, clear differences in ecological and sedimentological characteristics were found between two experimental areas (sheltered and exposed), subjected to slightly different hydrodynamic conditions. The one sheltered by coastal sandbanks was dominated by fine-muddy sand, higher species richness, biomass, and higher biological activity (burrows, fauna, and biological beds) as observed by all methods in one or another way. Moreover, functional diversity indices revealed a higher partitioning of the total available resources, suggesting more complex ecological processes in the sheltered area. Conversely, the area more exposed to the open sea was dominated by more sandy sediments, and fewer organisms were found. The combination of those different monitoring tools provides an integrated, complementary view, from different perspectives, on the biological, physical and functional characteristics of the study areas.

## Introduction

Over the last century, coastal countries have implemented hard engineering structures to maintain beach levels and resist exposure to severe climate events (Williams et al., 2018). Typically, groynes and seawalls are combined with beach nourishment schemes, and the association of both is the dominating form of coastal defence used worldwide (Spalding et al., 2014). However, while they have shown to be effective in some cases, they are also responsible for important financial costs due to maintenance and the necessity of sand replenishment every few years (Morris et al., 2018; Speybroeck et al., 2006; Temmerman et al., 2013; Williams et al., 2018).

Nature-based solutions are defined by the European Commission as ‘actions that address environmental, social and economic challenges simultaneously by maximising the benefits provided by nature (…) inspired by, supported by, or copied from nature’ (European Commission, 2015). They have gained increasing attention for coastal management strategies, as restoring or implementing natural coastal ecosystems could replace or complement artificial structures for coastal protection (Spalding et al., 2014; Temmerman et al., 2013). These ecosystems provide coastal erosion and flood defence services through ecological processes such as increased bottom-shear stress, localised water shallowing, sediment deposition and seabed stabilisation (Gracia et al., 2018; Speybroeck et al., 2006). Mangroves and coral reefs are very efficient at reducing coastal erosion, but they cannot be implemented in temperate regions. On the Atlantic European coastline, one example of an effective ecosystem to reduce wave height and protect the shoreline is salt marshes, but their distribution is limited as they have been drained since the Middle Ages for land reclamation or dikes’ construction (Cattrijsse & Hampel, 2006; Narayan et al., 2016). Seagrass meadows are also another efficient marine ecosystem for coastal protection and are present on the Atlantic European coastline (such as *Zostera marina* and *Zostera noltei*), but they are not present in Belgian waters (Ondiviela et al., 2014; Tullrot, 2009). Other solutions must therefore be considered to protect the sandy coastline of the Belgian coast. In the nearshore area, aggregations of the tube-dwelling polychaetes *Lanice conchilega* are forming aggregations on soft sediments (Degraer et al., 2008; Rabaut et al., 2009). Moreover, the reef-building blue mussel *Mytilus edulis* is also found in the Belgian part of the North Sea (thereafter named BPNS), most of the time colonising artificial hard substrates (Degraer et al., 2019). Although no established subtidal mussel beds on soft sediment have been found occurring in the area, its natural presence in Belgian waters suggests that the environmental conditions present in the BPNS are appropriate for mussel bed development.

The “Coastbusters” projects are a Belgian public-private partnership borne out of this need to develop sustainable and more environmentally friendly alternatives in coastal protection systems by using nature-based solutions. The first field pilot project (2017–2020) was set-up in the BPNS to test the feasibility of using reef-initiating structures for three types of bio-builder organisms (seaweed, blue mussel, *L. conchilega*) as nature-based coastal protection elements to stabilise the shoreline (Goedefroo et al., 2022; Sterckx et al., 2019). The resilience, survivability and reef-building capacity of those three taxa were explored. Preliminary results demonstrate a high potential for *M. edulis* beds, since a mussel bed developed during summer and the beginning of fall of 2019. Unfortunately, they mostly disappeared each winter season, probably due to predation (sea stars) and winter storms (Goedefroo et al., 2022).

Following these promising results, a follow-up project “Coastbusters 2.0” (2020–2023) is focused on optimisation of monitoring techniques of the mussel bed development area and on potential ecological responses to the newly created biogenic reef. In particular, to understand the impact of abiotic conditions on reef development, this project focuses on two areas with different hydrodynamics (sheltered and exposed).

To fully capture the ecological structure of the area, an integrated ecological monitoring program was indispensable. Therefore, traditional benthic assessment methodologies (grab sampling and granulometry) and 2 non-invasive benthic assessment techniques (sediment profile imaging and transect diving video surveys) were used for the baseline monitoring of the areas prior to the installation of the aquaculture set-up for facilitating mussel bed development. The sediment profile imaging (SPI) consists of a camera delivering undisturbed images of the water-sediment interface and the presence of biological structures (i.e. burrows, tubes) (Van Hoey et al., 2014). Past studies have proven the usefulness of the combined use of benthic grabs and SPI to provide an accurate assessment of the biodiversity and functioning of benthic systems (Birchenough et al., 2012; Van Hoey et al., 2014; Wilson et al., 2009). Additionally, video surveys have been widely used in the past as a tool for characterisation, impact assessment and monitoring of the seafloor (Fields et al., 2019; Karatayev et al., 2018; Sheehan et al., 2010). In this sense, most of the studies mentioned rely on towed sledges attached to cameras for filming the areas. However, irregular topography and variable visibility of the studied area make diving surveys a more suitable option for this study area, since the diver can adapt easily to challenging conditions while filming (Kendall et al., 2005). Furthermore, diving surveys are usually less destructive than some towed sledges that may cause disturbances to the seabed. All these techniques are not new, but using them in an integrated way is seldomly executed and should deliver an integrated view on the biological (species density, richness, diversity and biomass), biogeochemical (redox potential sediment layers) and functional (functional indices) characteristics of the soft-bottom ecosystems. This study is a baseline survey of the area where a biogenic reef (*M. edulis*) will be induced. This work allows us to assess the suitability, redundancy and added value of the combination of different monitoring methodologies and parameters obtained from them.

The research questions for this study are as follows: (1) Can a comprehensive understanding of the studied area’s habitat be achieved by combining quantitative and qualitative data from video surveys, SPI and Van Veen grabs? (2) Are variations in hydrodynamic conditions among the study areas reflected in the sedimentological and biological structural characteristics (density and diversity) of the benthic habitats? (3) Is this observed difference also reflected in the biogeochemical state and measures of functional diversity? The main objective of this study is to get an integrated perspective on the biological, physical and functional characteristics of the area. This integrated approach would serve as a robust foundation for the future longer-term monitoring of newly established biogenic reefs.

## Materials and methods

### Study area and experimental design

The BPNS is a small shallow area within the Southern Bight of the North Sea covering around 3600 km^2^, with an average depth of 20 m. Given the presence of multiple sandbanks, the area is characterised by a complex topography (Verfaillie et al., 2006) and highly variable benthic sedimentary habitats in terms of grain size (Breine et al., 2018; Van Hoey et al., 2004).

**Fig. 1:**
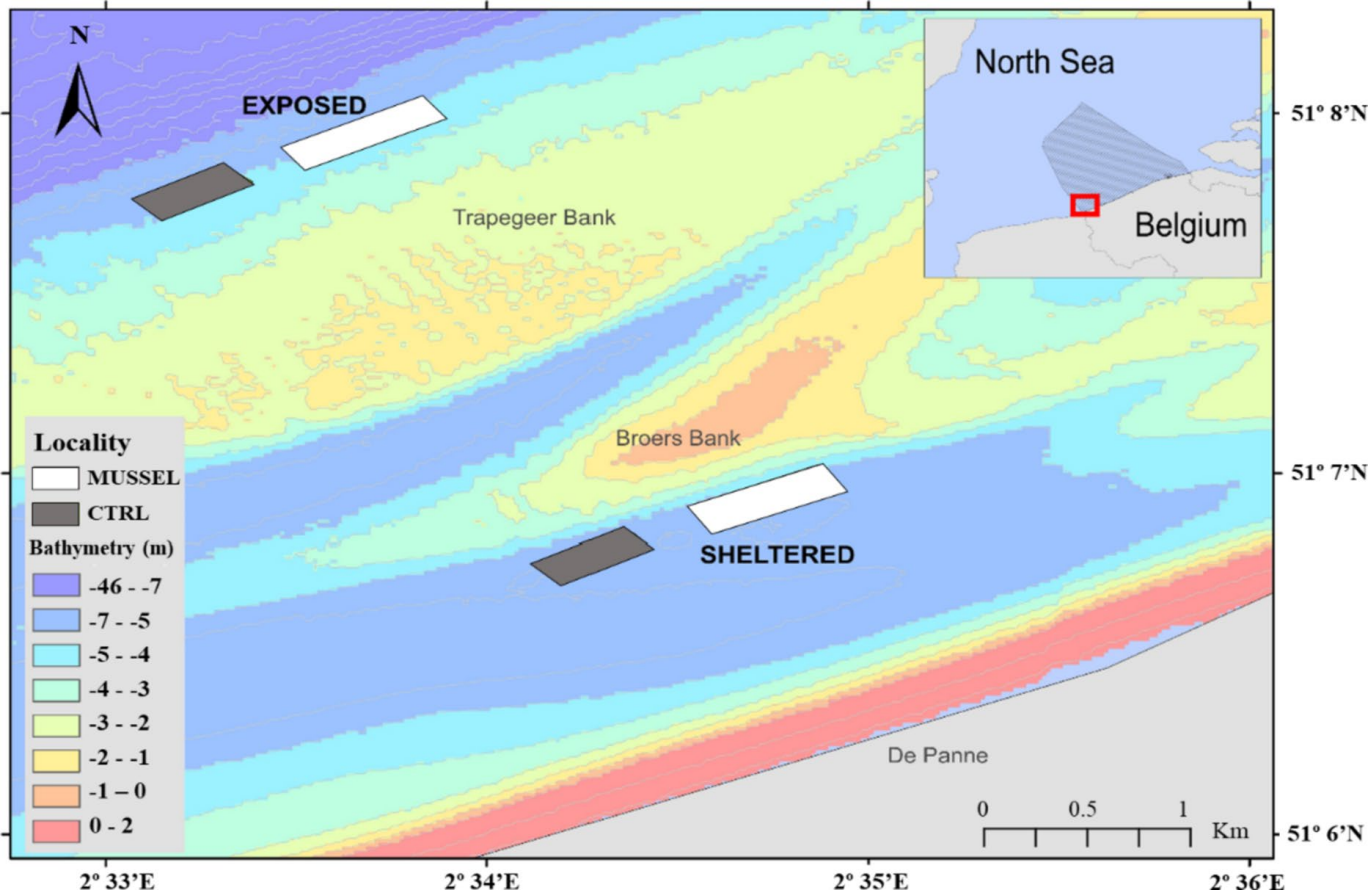
Bathymetry map and location of sampling sites. MUSSEL (mussel development area, area where mussel-growing longline system will be installed; 5 replicates); CTRL (control area; 5 replicates)

Within the BPNS, the “Coastbusters 2.0” experimental site is located in a subtidal area in front of the town of De Panne (Fig. 1). This study focuses on two separated areas under different hydrodynamic conditions: a sheltered (51° 07′ 19.2″ N, 2°35′ 16.8″ E) and an exposed area (51° 07′ 22.2″ N, 2° 33′ 28.5″ E). They lie respectively 2 and 5 km from the shore. The sheltered area is located south of the Broers sandbank, relatively sheltered from the waves and currents, and the exposed area is located north of the Trapegeer sandbank, facing the open sea (Fig. 9) (Langedock et al., 2020). The mean depth of both areas is around 5 m (lowest astronomical tide) (Fig. 4). A sea surface and bottom current estimate for both areas were made based on data from 01 June 2017 until 01 June 2020 from the open source COHERENS V1 (v8.7) model based on the harmonic tides and the wind forecast (Fig. 10) (Langedock et al., 2020). Based on those information, we considered one as a sheltered area because of the lower bottom currents and position on the slope of the bank (coastward). Exposed is the area with slightly higher bottom currents and positioned on the seaward side of the bank.

Along each area, two types of sampling localities were selected (“mussel” and “control” locality) (Fig. 1). The underneath and nearby (10 m^2^) areas of the ‘mussel’ locality potentially correspond to the future aquaculture longline and dropper line installation, which will support the creation of the mussel bed. A control locality is 500 m westwards from the installation, away from the dominant residual current direction and therefore out of the influence sphere (Fig. 1). Five replicates of SPI and Van Veen grabs were taken within each locality and area. Sampling was conducted along 2 campaigns in June and September 2020 (Table 1).

**Table 1:** Van Veen grab, SPI and video surveys sampling campaigns

Sampling campaign	SPI	Van Veen grab	Video survey
T0	16th June 2020	15th June 2020	15th and 26th June 2020
T1	10th September 2020	7th September 2020	7th and 8th September 2020

This design provides an effective basis for the future use of a before/after–control/impact (BACI) design (Underwood, 1992). However, we expect no current mussel/control (locality) or before/after effects in our results, since the sampling was conducted only prior to the installation of the aquaculture set-up. Still, this experimental design encompasses 3 factors. ‘Area’ is a fixed factor with 2 levels, and it seeks to examine differences between sheltered and exposed hydrodynamic conditions. ‘Locality’ factor is fixed, and it incorporates the potential mussel bed site against a control. ‘Campaign’ is fixed and refers to different sampling periods. Locality and campaign are factors intended to test the effect of the mussel bed formation in the future. However, some effects from the previous mussel bed formation in 2019 (only in the mussel-sheltered area) and seasonal effects may be expected (Goedefroo et al., 2022). All factors are orthogonal to each other.

### Sampling techniques and data analysis

#### Van Veen grabs

A total of 40 granulometry and macrobenthos samples were collected by means of a Van Veen grab (sampling surface of 0.1 m^2^). Before rinsing the Van Veen samples over the sieve, sediment samples for granulometric analyses were collected using a PVC tube (core of 5 cm diameter), stored and further processed in the lab. The grab samples were washed onboard over a 1-mm mesh sieve and fixed in an 8% formaldehyde-seawater solution with eosine for easier visual detection of the benthic organisms.

The sediment samples were analysed by laser diffraction (Malvern Mastersizer 2000) for sediment composition (the percentages of clay, silt, sand and gravel) and median grain size. The coarse fraction (> 1600 μm) was sieved off before laser diffraction. The determination of the percentage of total organic carbon (TOC) in the sediment samples was carried out using a modified Walkley-Black titration method (Gaudette et al., 1974).

Benthic organisms were identified to the lowest possible taxonomical level and counted. Taxa were weighed as wet weight (WW) biomass to the nearest 0.00001 g. Density and biomass were standardised to m^−2^. Abundance and biomass data from one sample in the mussel-sheltered site during the T0 sampling campaign was discarded from the analyses because of bad conservation of the organisms. Statistical analyses were performed on macrobenthic diversity, functional parameters and structure of the community.

Macrobenthic communities were described by means of biotic and diversity indices (density (N), biomass (B), species richness (S), Shannon index (H′(loge)) and Simpson index (1−λ′)). The functional characterisation was done by means of functional indices calculation: functional richness (FRic), functional evenness (FEve), functional divergence (FDiv), Rao’s quadratic entropy (RaoQ) and the community bioturbation potential (BPc) (Festjens et al., 2023). Those four indices were selected as they are considered complementary measurements of functional diversity attributes for different environments and assemblages (Gusmao et al., 2016). BPc was calculated according to the methodology proposed by Solan et al. (2004) and Queirós et al. (2013). In order to allow the comparison with previous research conducted in the BPNS, abundance and biomass data for BPc were used as count per sample (0.1 m^2^) and not standardised value per square metre (Breine et al., 2018). When the required information on sediment reworking (Ri) and mobility (Mi) of species was not defined, values from taxonomically close species were used. Ri and Mi values were obtained from Breine et al. (2018) and Queirós et al. (2013).

To determine whether area, locality and their interaction have an effect on the benthic characteristics and functional diversity indices, linear mixed models with area (two levels, exposed and sheltered) and locality (three levels, control and mussel) were chosen as categorical fixed effects and sampling campaign as a categorical random effect (two levels, T0 and T1). The normality and linearity of the residuals were tested by visual inspection of the residuals versus fitted values plot and with a Shapiro-Wilks test, and the homogeneity of variances was checked using Levene’s test. If these assumptions were not satisfied, data were transformed or the Kruskal-Wallis non-parametric test was used when no transformation managed to meet the assumptions (Table 5). Moreover, non-metric dimensional scaling (nMDS) built from a Bray-Curtis dissimilarity matrix based on abundance data was used to visualise the different macrobenthic community structures. A nMDS is an ordination method attempting to represent as closely as possible the pairwise dissimilarities between objects in a low-dimensional space. The stress value provides information on the quality of the ordination plot. A PERMANOVA was performed on the Bray-Curtis similarity matrix and followed by a permutational multivariate analysis of dispersion (PERMDISP) to check for multivariate spread among factors. A similarity of percentages (SIMPER) analysis was used to identify the most contributing taxa to the dissimilarities between exposed and sheltered areas. Analyses on community structure were calculated in PRIMER v6 software, while all the other analyses were performed with R Studio (version 1.4.1106) with the ‘FD’ package in R (Laliberte & Legendre, 2010).

#### Sediment profile imagery

A SPI device was used to obtain pictures of the water-sediment interface to provide biological, chemical and physical features of the localities for seafloor assessment (Birchenough et al., 2006, 2012). The device is composed of a frame and a core with a camera (Nikon D7200). When the SPI reaches the sea bottom, a lever activates the camera, and two pictures per sample station were taken: a first picture 10 s after activation of the lever and a second picture 15 s later, but only 1 of them will be analysed. In total, 40 pictures were analysed helped by SpiArcBase (Romero-Ramirez et al., 2013) and ImageJ (Schneider et al., 2012) softwares. Data was obtained for a set of 16 variables (Table 2). Median grain size class was estimated from a standardised set of pictures corresponding to a measured grain size that will be compared with grain size data obtained from laser diffraction of sediment samples (see the “Van Veen grabs” section).

**Table 2:** Overview of parameters obtained from the SPI technique, acronyms, method or software used and type of variable (N numerical, C categorical)

Parameter type	Parameters	Acronym	Obtention method	Type
Overall sediment features	**Mean penetration depth (**of the SPI device plate into de sediment); linked to sediment compactness (Rhoads & Germano, 1982)	MPD	SpiArcBase	N
	**Sediment-water interface (SWI) length**	SWI	ImageJ	
	Median grain size	MGS	Observation	C
Biogeochemical features	**aRPD (mm)**	RPD	SpiArcBase	N
	Maximum depth aRPD (mm)	mRPD	SpiArcBase	N
	SPI width	W	SpiArcBase	N
	**Percentage of anoxic sediment**	AA%	Calculation*^1^	N
	aRPD range	rRPD	Calculation*^2^	N
	**Length of the aRPD**	lRPD	ImageJ	N
Biological features	Presence (P.) of biogenic beds (mainly *L. conchilega*)	BIOB	Observation	C
	P. of surface fauna	SF	Observation	C
	P. of faecal Pellets	FP	Observation	C
	P. of feeding mounds or pits	FMP	Observation	C
	**No. infaunal organisms**	INF	Observation	N
	No. Burrows	BN	Observation	N
	No. Surface fauna (per species)	SF_n	Observation	N

In bold: variables used in the mixed multivariate matrix for FDMA analysis

*^1^AA% = anoxic sediment area/total sediment area*100

*^2^rRPD = |mRPD| − RPD*2

Based on some parameters obtained from the SPI pictures, the Benthic Habitat Quality (BHQ) index was calculated. This index was primarily developed for Scandinavian fjords (Nilsson & Rosenberg, 1997), but an adapted version was used in this study due to important differences in environmental conditions between Scandinavian fjords and the BPNS. In this adapted BHQ, the percentage of anoxic sediment is considered instead of the apparent redox potential discontinuity (aRPD) depth, scoring up to 5 points starting with 0%, then 1–25%, 25–50%, 50–75% and 75–100%. Presence of surface fauna, infauna and faecal pellets scores 1 point each. The number of tube worms and the number of burrows (any aRPD anomaly caused by a burrowing organism) account for up to 3 points each. BHQ consists of a 0–15 range index obtained from summing these assigned points. BHQ values greater than or equal to 6 indicate a “good” quality of habitat (Rosenberg et al., 2004).

Univariate statistical analyses were conducted on all studied variables. Additionally, a factor analysis of mixed data (FAMD) was performed based on a mixed multivariate matrix including some of the variables assessed from SPI (Table 2). This multivariate analysis allows us to assess the proportion of the variance explained by each variable and the similarity between observations. FAMD was performed in R Studio with “FactoMineR” and “factoextra” packages. In order to handle missing data, an imputation method was performed through a PCA method (“missMDA” package), so missing values were filled in all variables.

#### Seafloor characterisation

Diving surveys were recorded to monitor seafloor characterisation as in Goedefroo et al. (2022). The start and end filming points by the diver were georeferenced. The diver slowly moved forward and recorded through a 50-m (100 m in case of T0 mussel localities) transect following a guided rope marked with a black dot every metre. In total, 16 transects were recorded: 8 for each sampling campaign (Table 1), in which 4 corresponded to each area (sheltered and exposed), 2 transects in each locality (mussel and control).

Each metre from the video survey footage was documented through screenshots (this was done manually by pausing the video and using the marked rope as guidance). Due to poor visibility and suboptimal image quality, modifications to colour, contrast and brightness were applied using the “Auto enhancement 1” setting in ImBatch software version 7.3.0. The images were subdivided into 8 equal quadrats. Subsequently, every quadrat was assessed through six categories (Table 3), and the number of quadrats of each category was used to categorise each image into classes (Table 4). Finally, the rest of the classified screenshots were georeferenced by first interpolating the coordinates along the transect line between the starting point and the endpoint and then applying a 0.5 m perpendicular offset to the appropriate side of the line. The resulting matrixes were imported into Geographic Information System, ArcGIS® 10.4.1., ESRI as point data with the objective of visualising the seafloor features on a map.

**Table 3:** Categories for quadrat assessment

Category	Unit
Mussel cover	No. of quadrats (maximum of 8)
*L. conchilega* cover	No. of quadrats (maximum of 8)
Shell's cover	No. of quadrats (maximum of 8)
Sand cover	No. of quadrats (maximum of 8)
Unknown quadrats	No. of quadrats (maximum of 8)
Confidence level*^1^	High, medium, low (depending on quality of the footage)

*^1^Confidence level was used as a subjective categorical variable assessing the quality of the image and therefore the confidence in identifying structures in them. Quadrats with low confidence levels were considered unknown

**Table 4:** Decision table for image classification

Class	Sand cover	Shells cover	*L. conchilega* cover	Mussels cover	Unknown
Unknown (UN)	Irrelevant	Irrelevant	Irrelevant	Irrelevant	**>= 4 quadrats**
Bare sand (BS)	**≥ 6 quadrats**	Not used	None	None	Irrelevant
Bare sand with shells (BSS)	**> 6 quadrats**		None	None	Irrelevant
Shell dominated sand (SDS)	Irrelevant	**≥ 6 quadrats**	None	None	Irrelevant
Lanice Sparse (LS)	Irrelevant	Irrelevant	**≤ 2 quadrats**	None	Irrelevant
Lanice Patchy (LP)	Irrelevant	Irrelevant	**2–6 quadrats**	None	Irrelevant
Lanice Dominated (LD)	Irrelevant	Irrelevant	**≥ 6 quadrats**	None	Irrelevant
Mussel Sparse (MS)	Irrelevant	Irrelevant	Irrelevant	**≤ 2 quadrats**	Irrelevant
Mussels Patchy (M)	Irrelevant	Irrelevant	Irrelevant	**2–6 quadrats**	Irrelevant
Mussel Dense bed (MD)	Irrelevant	Irrelevant	Irrelevant	**≥ 6 quadrats**	Irrelevant

When conditions for no. of quadrats (in bold) were met, the picture was categorised as such. “Unknown” class corresponds to the impossibility of seabed feature’s identification (more than 4/8 unknown quadrats)

## Results

### Biotic parameters

A total of 93 taxa were identified from the Van Veen grabs for the two sampling campaigns with 6 different taxonomic groups: 32 taxa of Malacostraca, 42 taxa of annelids, 14 taxa of molluscs, 3 taxa of echinoderms, 1 taxon of Anthozoa and 1 taxon of Sipuncula. Except for the Shannon-Wiener index, all the structural indices were significantly different between exposed and sheltered areas (Table 5), while no significant differences could be found between mussel and control localities. Abundance (8288.42 ± 1529.84 ind.m^−2^), species richness (26.74 ± 2.27 spp.sample^−1^) and biomass (508.51 ± 142.52 g.m^−2^) had higher values in the sheltered area compared to the exposed area (3299.00 ± 792.05 ind.m^−2^; 17.05 ± 0.77 spp.sample^−1^; 100.37 ± 32.62 g.m^−2^). Only the Simpson index (1-D) had higher values in the exposed area compared to the sheltered area (Table 5).

**Table 5:** Structural biological indices, mean values ± standard error for each area (EXP and SHL) and associated *p*-values

Variable	EXP	SHL	Statistical test used	*p*-value
Abundance (ind.m^−2^)	3229.00 ± 792.05	8288.42 ± 1529.84	Linear mixed model (log transformation)	0.0005626*
Burrows (SPI)	1.4 ± 2.23	6.2 ± 6.07	Kruskal-Wallis	8.60E−06*
Species richness (spp.sample^−1^)	17.05 ± 0.77	26.74 ± 2.27	Linear mixed model (log transformation)	0.0003802*
Shannon-Wiener (H′)	1.78 ± 0.069	1.53 ± 0.12	Linear mixed model	0.07834
Simpson index (1-D)	0.73 ± 0.020	0.57 ± 0.043	Linear mixed model (Cube transformation)	0.004731*
Biomass (g.m^−2^)	100.37 ± 32.62	508.51 ± 142.52	Linear mixed model (log transformation)	0.0004217*

*p < 0.05

**Fig. 2:**
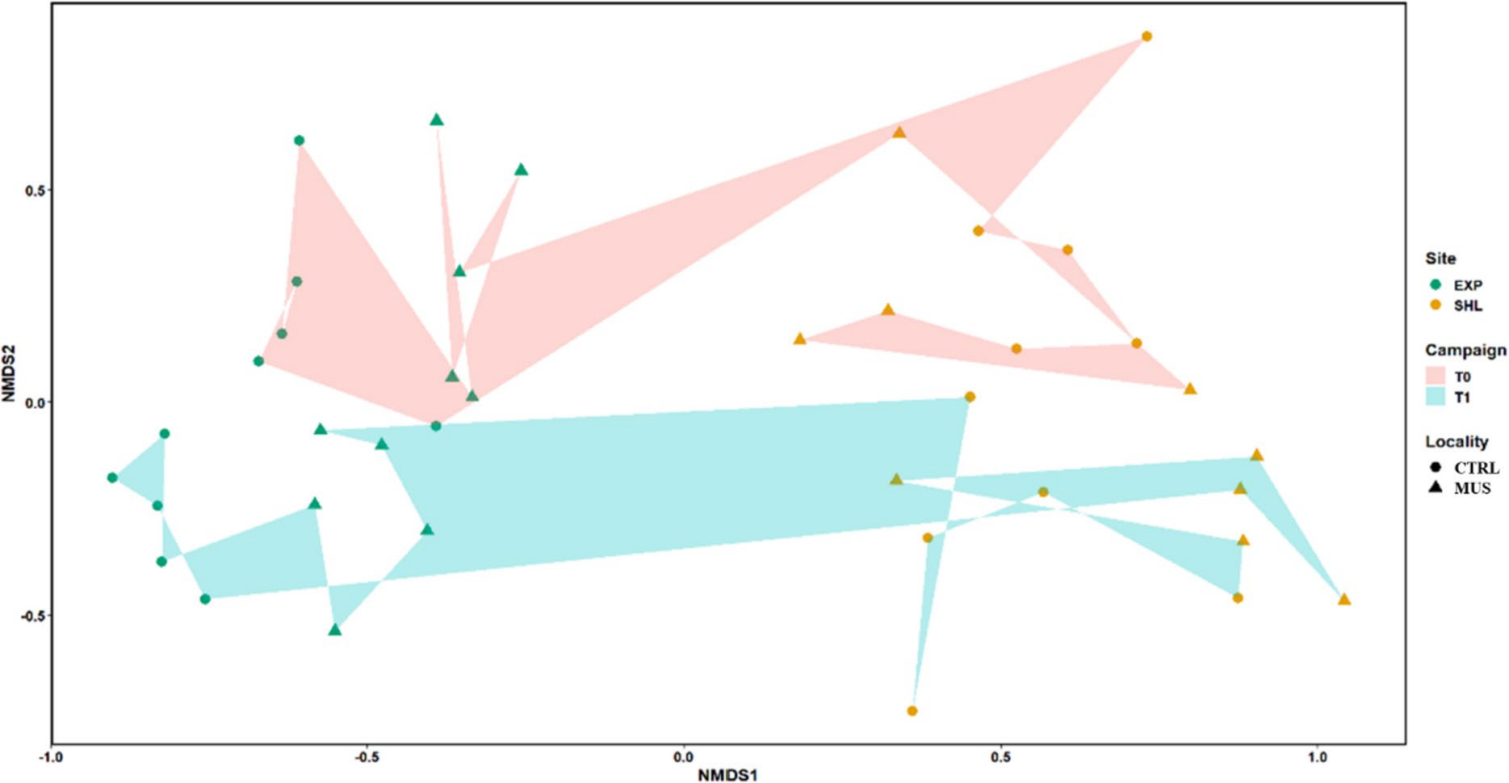
Non-metric multidimensional scaling (nMDS, performed on Bray-Curtis dissimilarity matrix) of macrobenthos abundance data separated by localities (MUSSEL, CONTROL), areas (EXP, SHL) and campaign (T0, T1). Stress value: 0.17

The nMDS plot (Fig. 2) represents the macrobenthic community structure and shows a clear difference between exposed and sheltered areas, as well as between the two sampling campaigns (T0, T1). However, no differences could be seen between the mussel and control localities. The PERMANOVA test revealed that the area (*R*^2^ = 0.40121, *p* = 0.001) and, to a lesser extent, the sampling campaign (*R*^2^ = 0.07465, *p* = 0.001) have a significant effect on the community structure. The *R*^2^ values show that the area was the factor having the biggest contribution (40.12%) to the variance. The top five contributing taxa to the dissimilarities between exposed and sheltered areas (EXP and SHL) were Oligochaeta spp., *Spiophanes bombyx*, *Magelona johnstoni*, *Magelona* juveniles and Cirratulidae, while Oligochaeta spp., *S. bombyx*, *Magelona* juveniles, *M. johnstoni* and *Lanice* spp. were the top 5 taxa contributing to the dissimilarities between sampling campaigns. They contributed 69.8% to the dissimilarity between the two areas, while 67.7% contributed to the dissimilarity between the two sampling campaigns.

**Fig. 3:**
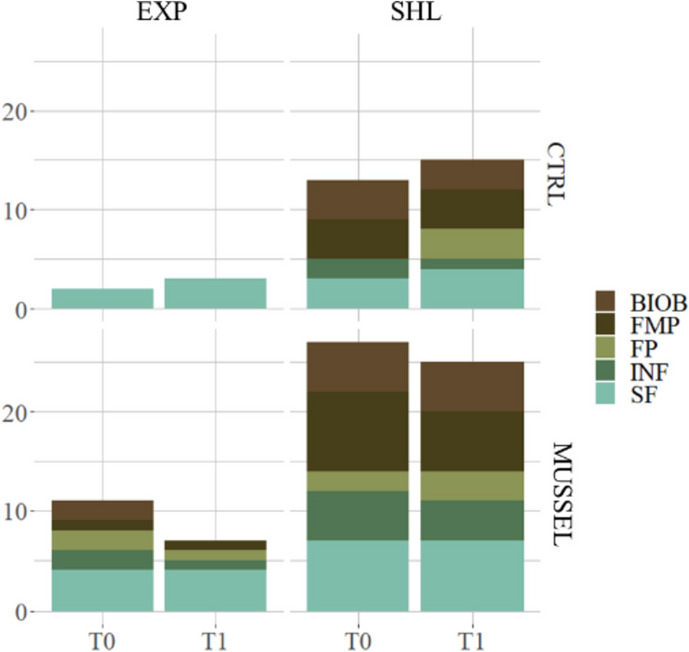
Cumulative occurrence of biological features found through SPI at each area, along both campaigns and localities. BIOB biogenic Beds, FMP feeding mounds or pits, FP faecal pellets, INF infaunal organisms, SF surface faunal organisms

Concerning biological parameters obtained through SPI, 22.5% of the pictures showed infauna, and 55% showed surface fauna embracing 7 different taxa, mainly *L. conchilega* but also the sea star *Asterias rubens* and the decapod *Crangon crangon*. Infauna and surface fauna, as well as other faunistic parameters such as faecal pellets, biogenic beds and feeding pits/mounds, were more frequently observed in the sheltered area (Fig. 3).

### Sedimentology and seafloor characterisation

**Fig. 4:**
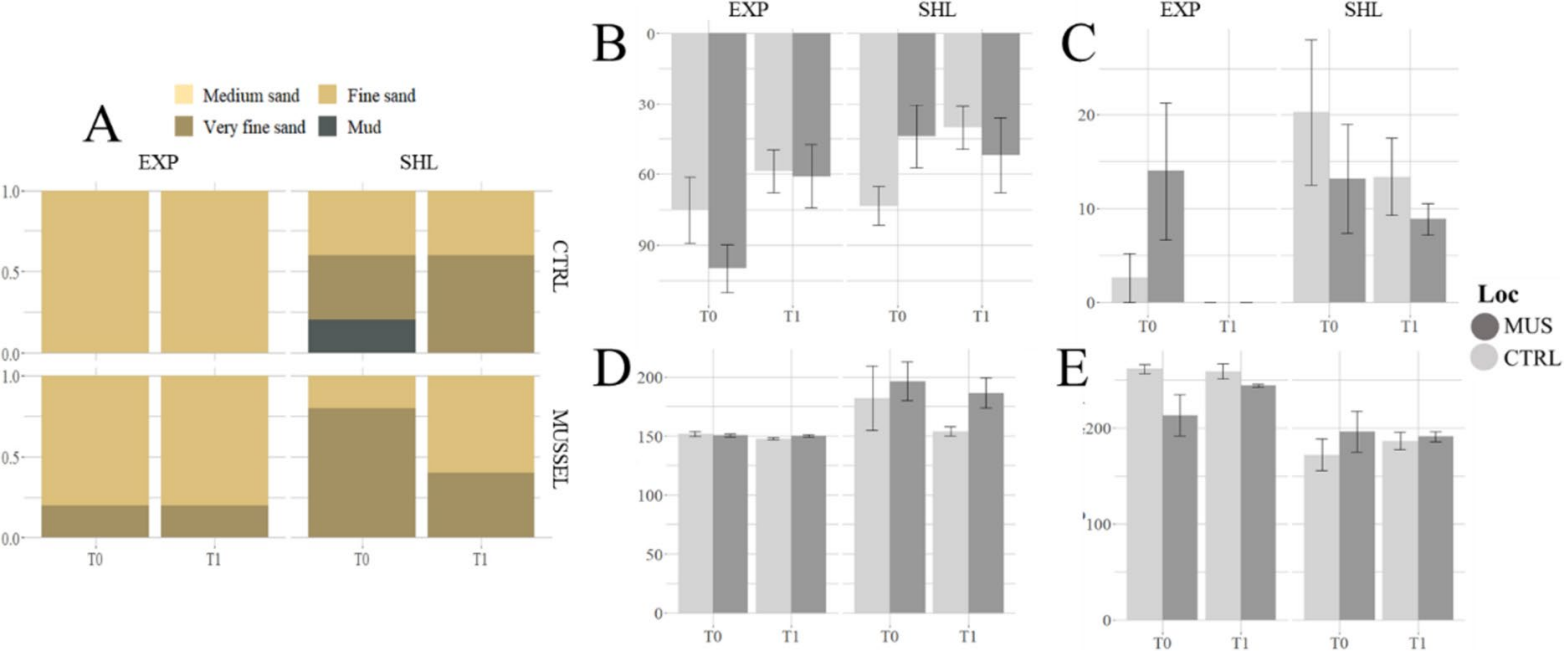
Sedimentological features at the exposed and sheltered areas for each locality and the two sampling campaigns. **A** Proportion of the median grain size observed through SPI. **B** Penetration depth of SPI (cm). **C** Mud content (0.01 μm < grain size < 63 μm). **D** Sediment-water interface (SWI) length. **E** Median grain size (μm). Vertical error bars represent standard errors

Sedimentological analyses based on the Van Veen grab samples showed a coarser median grain size in the exposed area (275.33 ± 6.75 μm) than in the sheltered area (196.11 ± 6.77 μm) (significant difference, *p* = 2.973E−07) and were relatively similar among localities in the same area. The median grain size reached its maximum and minimum at T0 in the control locality, respectively, in the exposed area (261.37 ± 4.40 μm) and in the sheltered area (168.56 ± 14.88 μm). Similar results were obtained through SPI assessment, where grain size exhibited a clear dominance of “very fine sand” throughout the sheltered, while the exposed one was homogenously dominated by “fine sand”. In addition, a much higher mud content was found in the sheltered area compared to the exposed area. Mud content has an additional seasonal change, with higher mud content present in T0 compared to T1. The maximum value was reached at T0 in the control locality in the sheltered area (20.27 ± 7.77%), where mud was also detected through SPI. However, no mud content (0%) was found for the two localities at T1 in the exposed area (Fig. 4C) (Table 6). TOC was significantly higher in the sheltered area compared to the exposed area but was not influenced by the locality (Table 6, Fig. 11). The compactness of the sediment can be assessed through SPI mean penetration depth, which also varied between areas, reaching significantly deeper layers in the exposed samples (73.72 ± 29.52 cm) compared to sheltered (52.40 ± 27.87 cm). Similar differences were detected in the length of the sediment-water interface (SWI) (Fig. 4).

**Table 6:** Mean values ± standard error of variables used to characterise the sediment features, statistical test used in the factor “area” and *p*-value

Variable	EXP	SHL	Test used for area	*p*-value
Penetration depth (cm)	73.72 ± 29.52	52.40 ± 27.87	3-way ANOVA	0.0164*
SWI length (cm)	149.9 ± 3.55	178.18 ± 43.11	3-way ANOVA	0.00753*
Mud content (%)	4.60 ± 2.18	14.64 ± 2.56	Kruskal-Wallis	7.193E−05*
Median grain size (μm)	275.33 ± 6.75	196.11 ± 6.77	Linear mixed model	2.973E−07*
TOC (%)	0.26 ± 0.05	0.50 ± 0.06	Kruskal-Wallis	0.0001075*

*p < 0.05

**Fig. 5:**
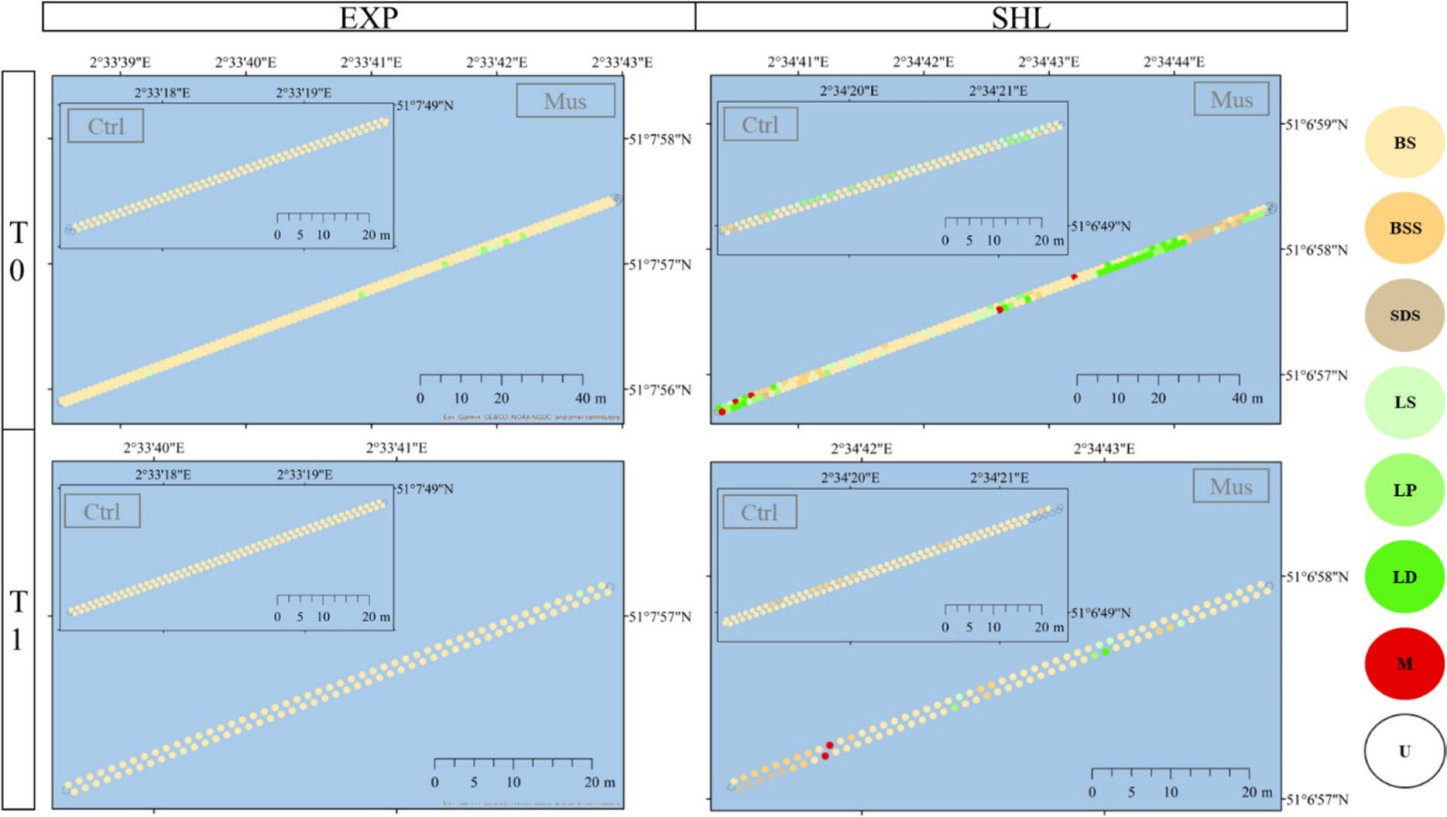
Seafloor characterisation of the Coastbusters areas in T0 and T1 through video snapshots analysis. Legend corresponds to variable “Class” (Table 5): unknown (U), bare sand (BS), bare sands with shells (BSS), shell dominated sand (SDS), Lanice sparse (LS), Lanice patchy (LP), Lanice dominated (LD), Mussel patchy (M)

With regard to sediment composition, video transects revealed that the top of the sea floor was sometimes covered by bivalve shells (SDS), especially in the sheltered area. However, the most common category was bare sand (BS) (Fig. 5).

Video transects also revealed that, in T0, some *M. edulis* patches were present in the sheltered “mussel” locality. This same site was also covered by dense *L. conchilega* aggregations, also found in the exposed “mussel” area. In T1, presence of mussel patches and *L. conchilega* beds was remarkably lower compared to the previous campaign. Highest occurrence of *L. conchilega* aggregations was found at the sheltered control transect (Fig. 5).

### Functional and biogeochemical characteristics

All the functional indices were significantly different between exposed and sheltered areas, while no significant differences could be found between mussel and control localities. Functional richness, functional evenness, functional divergence, Rao’s quadratic entropy, community bioturbation potential and BHQ had higher values in the sheltered area compared to the exposed area (Table 7).

**Table 7:** Functional indices and SPI biogeochemical parameters

Variable	EXP	SHL	Statistical test used	*p*-value
FRic	5.80 ± 0.36	8.44 ± 0.57	Linear mixed model	0.0007811*
FEve	0.49 ± 0.019	0.61 ± 0.014	Linear mixed model	7.083E−05*
FDiv	0.76 ± 0.019	1.44 ± 0.042	Kruskal-Wallis	0.00002*
RaoQ	0.39 ± 0.022	0.49 ± 0.036	Linear mixed model	0.02907*
BPc	118.32 ± 15.75	398.48 ± 89.25	Linear mixed model (log transformation)	0.006113*
RPD depth	44.87 ± 33.51	31.80 ± 26.67	3-way ANOVA (square root transformation)	0.285
RPD length	231.03 ± 52.16	289.38 ± 131.79	Kruskal-Wallis	0.298
BHQ	4.95 ± 1.19	7.55 ± 3.11	3-Way ANOVA	0.00241*

Mean values ± standard error for each area (exposed and sheltered), used statistical test and associated *p*-values

*FRic* functional richness, *FEve* functional evenness, *FDiv* functional divergence, *RaoQ* Rao’s quadratic entropy, *BPc* community bioturbation potential, *BHQ* benthic habitat quality index

*p < 0.05

**Fig. 6:**
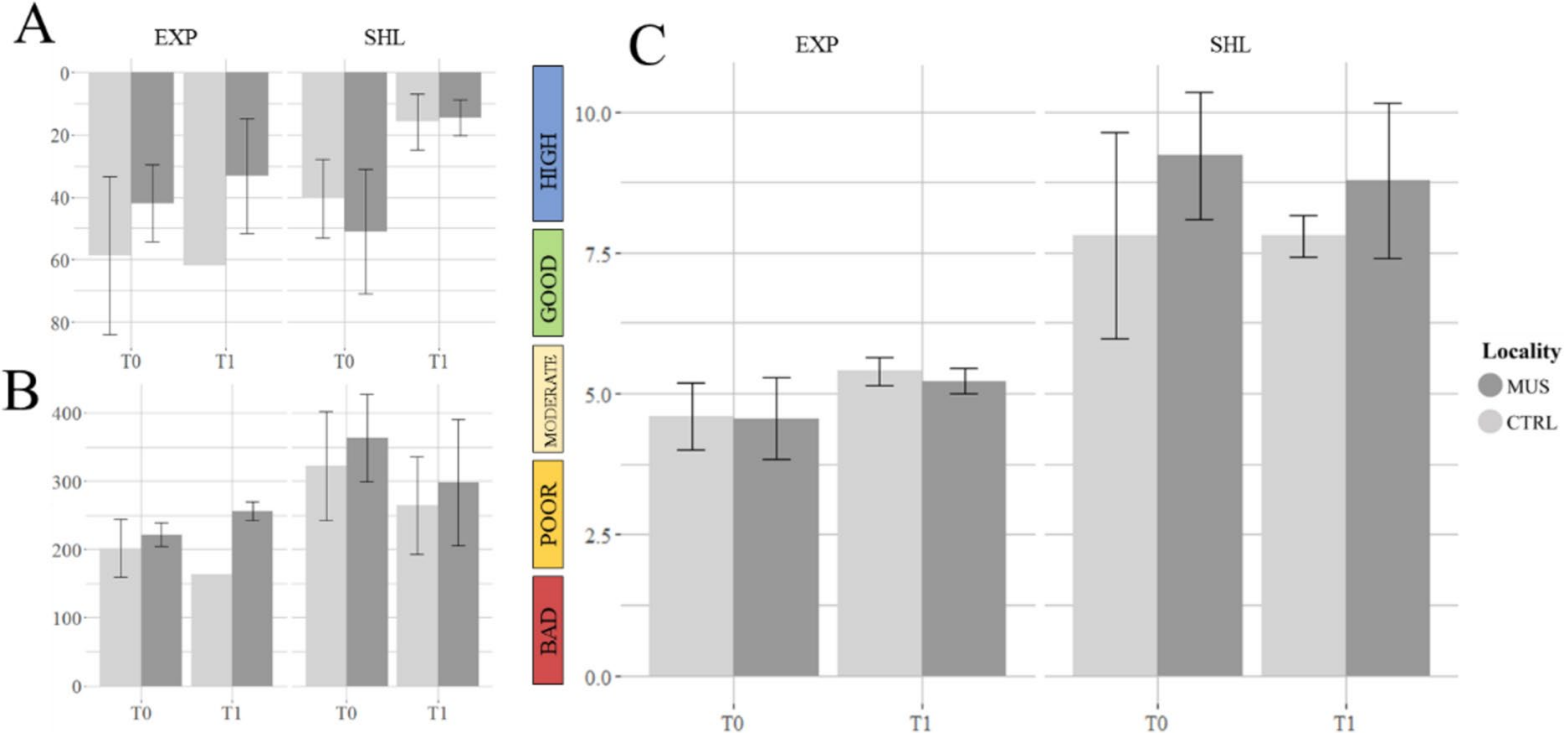
Biogeochemical features at the exposed and sheltered areas for each locality and the two sampling campaigns (**A** aRPD mean depth, **B** aRPD length) and **C** BHQ index (colour categories as in Nilsson & Rosenberg, 1997)

In relation to biogeochemical parameters obtained from SPI, only 17 out of the 40 analysed pictures (42.5%) exhibited an observable redox potential discontinuity layer (aRPD; 45% in the sheltered area and 40% in the exposed). In the remaining samples, only oxidised sediment was visible, meaning that the anoxic layer of sediment laid below the SPI penetration depth. In relation to oxic-anoxic boundary layer complexity, aRPD length was higher in the sheltered area compared to the exposed area (Fig. 6), but not statistically different. Mean depth of the aRPD did not show any significant difference either (Table 7).

Benthic habitat quality of the experimental area was, on average, good. BHQ index (> 6) was given to 32.5% of the samples. Sheltered sites obtained significantly higher BHQ index in comparison to exposed ones. Additionally, some other non-significant differences between mussel and control localities (only within the sheltered area) are identified, with higher BHQ values in the mussel locality compared to the control locality (Fig. 6C).

**Fig. 7:**
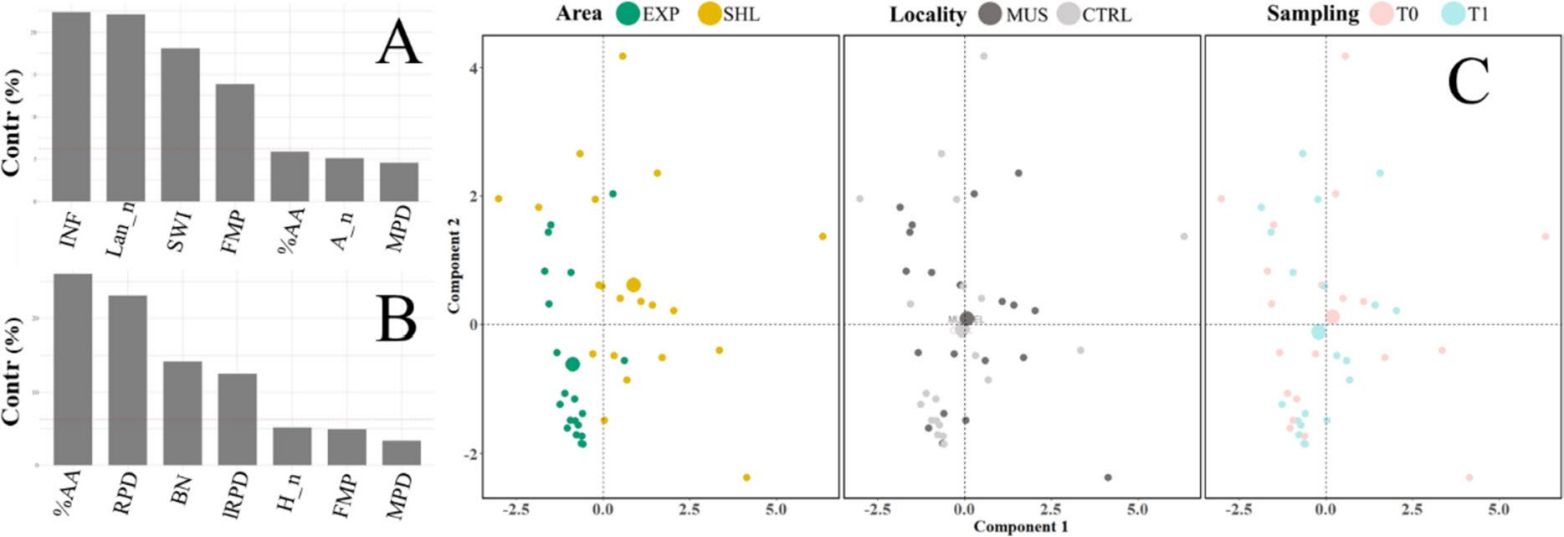
Variable contribution to components 1 (**A**) and 2 (**B**) of the multivariate analysis FAMD. Scatter plot of sampling points by area, locality and sampling through principal components 1 and 2 (**C**). Centroid (mean distance values) of the sample groups are in bigger size. Acronyms of variables are INF infaunal organisms, Lan_n number of *L. conchilega*, SWI sediment-water interface, FMP presence of feeding mounds or pits, %AA percentage of anoxic sediment, A_n number of *Asterias rubens*, MPD mean penetration depth, RPD aRPD, BN number of burrows, IRPD length of the aRPD, H_n number of hidroids

Results of the multivariate analysis (FAMD) on the SPI parameters showed that infauna and the number of *L. conchilega* were the variables that contributed most to component 1 (Fig. 7A), while the percentage of anoxic sediment and aRPD mean depth contributed most to component 2 (Fig. 7B). Samples located positively along component 1 mainly correspond to the sheltered samples, while exposed ones have negative values. Conversely, there is not a strong polarisation along component 2 (*y*-axis) (Fig. 7C).

## Discussion

To fully capture the dynamic of a newly established mussel bed and its influence on seafloor habitat and associated benthic communities, project Coastbusters 2.0 has included an important research component on the development of an integrated, holistic and scientifically advised monitoring strategy to follow up the research areas. In this study, we implemented traditional benthic assessment methodologies (Van Veen grabs and granulometry) and 2 non-invasive techniques (SPI, video diving surveys) for this. This combination of techniques has delivered an integrated view of the biological (species density, richness, diversity, biomass), biogeochemical (oxidised and anoxic layers), sedimentological (grain size, mud, habitat/community type [mussel bed, *L. conchilega* aggregations]) and functional (functional indices) characteristics of the sea bottom. Past studies have proven the usefulness of the combined use of benthic grabs and sediment profile imagery to provide an accurate assessment of the biodiversity and functioning of benthic systems, as well as the response of those systems to disturbances (Birchenough et al., 2012; Van Hoey et al., 2014; Wilson et al., 2009). This study also allows determining the initial ecological quality status of soft-sediment benthic habitats under different hydrodynamic conditions, which will be later subjected to a longline mussel aquaculture technique to establish subtidal reefs.

### Sediment properties and benthic communities along areas with different hydrodynamic conditions

Previous studies have indicated that low hydrodynamic conditions (Rodil et al., 2007) can favour the settlement of fine sediments in soft bottoms, while high hydrodynamics enhance sediment diffusion in the sediment-water interface. This aligns with the predominant sediment grain size observed in the present study (through multiple methodologies), where finer sediments were more prevalent in the sheltered area, particularly during summer (T0) (Fig. 4A, Table 6). SPI’s penetration depth is typically associated with the compactness/permeability of the seabed (Rhoads & Germano, 1982), which was more compact (less water content) in the sheltered area (muddy bioturbated sediment) compared to the exposed (sandy stirred sediment) (Fig. 4B). In this context, existent higher flow conditions at the exposed area (Fig. 9, Langedock et al., 2020) seem to be influential on the sediment type in the study area.

However, water flow is not the only direct variable influencing sediment features. Occurrence of *L. conchilega* aggregations and spare mussel patches, especially in sheltered localities (Fig. 3), enhances sedimentation of particles and also detritus, creating mounds (resulting in high values of SWI) (Rabaut et al., 2007; Van Hoey et al., 2008). Therefore, muddier sediments can be indicative of organically enriched sediments, which determine the community inhabiting soft bottom ecosystems (Robertson et al., 2015; Ysebaert et al., 2009). This is confirmed by TOC results which, as mud content, was higher in the sheltered area compared to the exposed area. This may account for the dominance of detritus-feeder species (e.g. Oligochaeta) observed in the sheltered area, and its lower Simpson index values compared to the exposed area (Goedefroo et al., 2022). This result suggests the linkage between the hydrodynamic conditions and occurring sediment type, but never disregarding the role of benthic communities (Foulquier et al., 2020; Van Hoey et al., 2004).

Differences in the macrofaunal communities’ characteristics of both sheltered and exposed areas are evident in variables determined by SPI methodology and Van Veen sampling. Hydrodynamic conditions, a recognised key descriptor for ecological richness (Van Colen et al., 2010; van der Wal et al., 2017; Ysebaert et al., 2003), enhanced higher density, species richness and biomass values in the sheltered area, typically low flow environment. Consistently, parameters qualitatively assessed through SPI indicate more frequent evidence of biological activity in the sheltered area. However, the two diversity indices assessed in this study were not similarly impacted by the hydrodynamic conditions. While the Shannon index did not show any significant difference between exposed and sheltered areas, the Simpson index had higher values in the exposed area (Table 5). First, the lack of significant differences in the Shannon index can be explained by the fact that, for a site with high species richness and low evenness, it may yield the same index value as a site with low richness but high evenness. Conversely, the Simpson index incorporates measures of evenness and species richness but gives greater weight to abundant species and is less sensitive to rare species than the Shannon index. Therefore, high evenness values found in exposed areas might be explained by the dominance of some taxa in the sheltered area, such as Oligochaeta.

The structural responses of infaunal assemblages from Van Veen’s methodology were undeniable. Even though other factors also had an influence on the community composition, the area was the factor contributing the most to the dissimilarities between stations at the nearshore and offshore areas (40.12% based on the PERMANOVA). Although less pronounced, this pattern is consistent through multivariate analysis (FAMD) of SPI parameters, especially since the clustering of sheltered and exposed areas was mainly driven by the presence of infauna and the bio-irrigator *L. conchilega* (Fig. 7). Results brought by these two techniques align and complement each other for the characterisation of sediment-dwelling communities.

### Eco-functional and biogeochemical characteristics of soft-bottom ecosystems along areas with different hydrodynamic conditions

In order to evaluate environmental quality beyond the scope of biodiversity, various functional indices have been widely used in marine ecology (Rosenberg et al., 2004; Van Hoey et al., 2013). One important biological trait influencing soft-bottom ecosystem functioning is bioturbation (Biles et al., 2002), favouring nutrient exchange in the sediment-water interface (Breine et al., 2018; Queirós et al., 2013; Volkenborn et al., 2007). BPc exhibited significantly lower values in the exposed area than in the sheltered area. These findings suggest reduced benthic-pelagic fluxes and nutrient cycling in the exposed area (Gusmao et al., 2016; van der Wal et al., 2017).

**Fig. 8:**
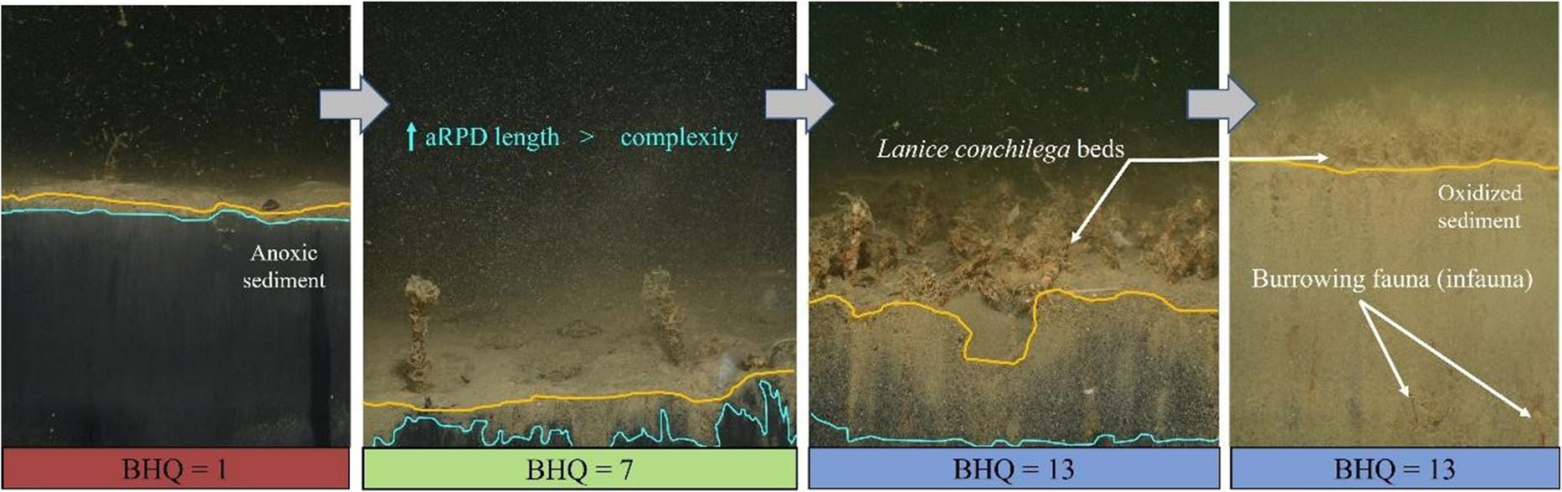
Succession of pictures taken through SPI in this study with increasing habitat quality index (BHQ). aRPD (blue line) and SWI (orange line) are represented

**Fig. 9:**
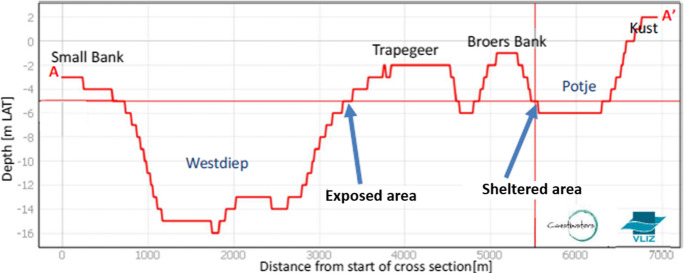
Cross section of the bathymetry from 7 km offshore at Small Bank (A) to the coastline of De Panne (A′). Depths in metres of lowest astronomical tide. Sandbanks are marked in black, channels are marked in blue and locations of both areas are marked in bold and indicated by a blue arrow. Modified from Langedock et al. (2020)

**Fig. 10:**
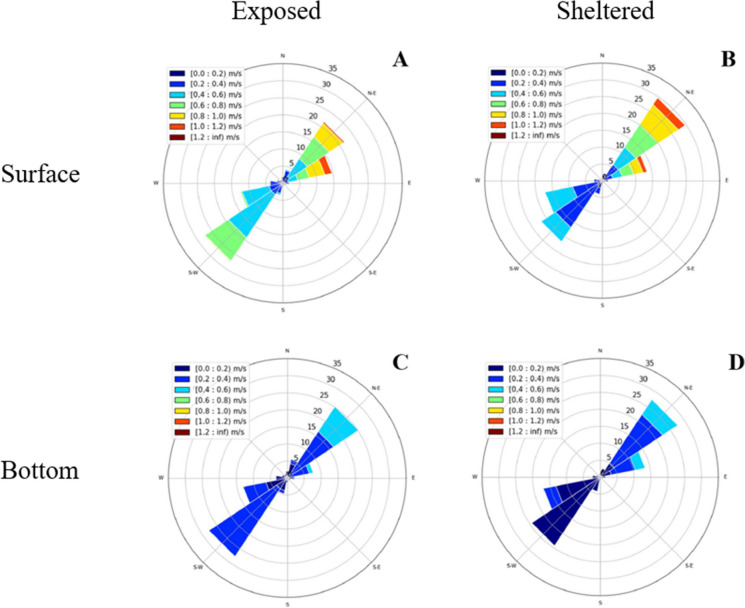
Surface and bottom currents at exposed (**A**, **C**) and sheltered (**B**, **D**) areas. Concentric circles indicate the percentage of the time the current runs in a specific direction. The colours of the vectors represent the magnitude. Due to bottom friction, the modelled current magnitude at the bottom (**C**, **D**) is approximately half the current magnitude at the surface. While the average modelled NE-going surface currents were stronger in the sheltered area, the average modelled NE-going bottom currents are stronger in the exposed area. Modified from Langedock et al. (2020)

**Fig. 11:**
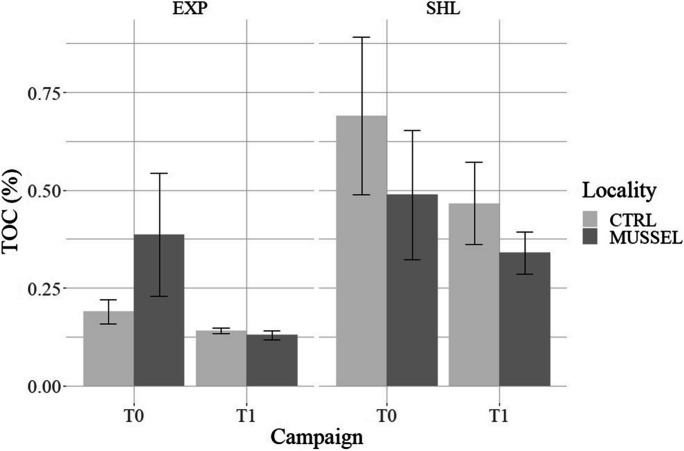
Mean organic enrichment (TOC) values at the exposed and sheltered areas for each locality and the two sampling campaigns. Vertical error bars correspond to standard error

Oxygenation of the sediment is typically favoured by high bioturbation potential. In this context, aRPD is often related to BPc values, with deeper aRPDs associated with higher BPc values (Birchenough et al., 2012). However, in our study area, aRPD depth was only slightly higher in the sheltered area (although without significance) (Table 7). This minor inconsistency could be explained by occasional strong sedimentation processes along the exposed area, resulting in a thick top layer of oxidised sediment that would bury the anoxic layer deeper than the SPI range. The length of the aRPD was greater in the sheltered area compared to exposed areas (Fig. 6, Table 7) although not statistically significant. Similarly, the frequency of “burrows” was higher. These two parameters are interconnected, as burrows are defined as any vertical irregularity or displacement in the sediment layers potentially caused by biological activity (Nilsson & Rosenberg, 2000). This pattern of anoxic sediment in the sheltered area suggests that higher biological activity indexes (such as BPc) may be linked to higher three-dimensional complexity of the oxic-anoxic boundary layers, not only to its mean depth (Fig. 8). This supports the concept of a three-dimensional pattern of oxidised sediment throughout the reduce layer linked to soft-bottom habitats, as suggested by Nilsson and Rosenberg (2000).

Higher frequency of *L. conchilega* beds and infauna was found in the sheltered area. Several authors have reported a correlation between greater habitat quality and high densities of *L. conchilega* (Rabaut et al., 2007; Van Hoey et al., 2008). These bio-builder polychaetes increase the structural complexity of the seafloor, creating niches that can be utilised by species with different functional attributes (Rabaut et al., 2009). Therefore, *L. conchilega* aggregations (Figs. 3 and 4) may enhance biological diversity and activity, thereby increasing habitat quality in the sheltered area. Although benthic habitat quality was predominantly “good” (32.5% of the samples), the BHQ index was significantly higher in the sheltered area compared to the exposed.

The overall functional diversity was approximated by the indices’ functional richness (FRic), functional evenness (FEve), functional divergence (FDiv) and Rao’s quadratic entropy (RaoQ). They all behaved similarly to the hydrodynamic area differences and indicated lower values in the exposed area than in the sheltered area. As FRic does not include information on relative abundances and is positively correlated with species richness, we found the same pattern as species richness which also had higher values in the sheltered area, which was expected (Llanos et al., 2020). The lower FEve in the exposed area indicates that some parts of the niche space are under-utilised, indicating some redundancy in the functional traits among species and competition. A non-optimal use of the resources decreases productivity and therefore food web support to higher trophic levels (Mason et al., 2005). The lower functional divergence in the exposed area indicates assemblages with a lower relative abundance of species with extreme categories of functional traits (Gusmao et al., 2016). Therefore, this corroborates the results from FEve as it also suggests a lower niche differentiation in the exposed condition compared to the sheltered condition and then more resource competition in strong hydrodynamic conditions (Mason et al., 2005). RaoQ is a measure based on the relative abundance of species in a community and some measure of trait dissimilarity among them. This index is highly correlated with the Simpson index, but opposite results were found in this study. This is because the Simpson index used was 1-D which has the preferred property to increase with greater diversity than the Shannon index. The Simpson index λ is the form used in RaoQ, explaining the opposite results for those two indices. The lower RaoQ values in the exposed condition suggest a community with low trait differentiation and low species abundance. The latter is confirmed by the analysis of the density which had much lower values in the exposed area. The integrated analysis of the four complementary functional diversity indices revealed a lower functional diversity in the exposed area compared to the sheltered area. The main conclusion is that there is better resource-use efficiency in the sheltered area and therefore probably a more valuable ecosystem functioning (Cadotte et al., 2011).

### Considerations on methodology

The implementation of nature-based solutions requires a rigorous environmental impact assessment to ensure that no damage is caused to the existing habitat and associated communities or that the desired habitat changes (nature-based solutions) are obtained. Yet this involves the implementation of methodologies that can still be improved. For example, regarding SPI utilisation, some authors suggest that future studies using the SPI camera need to acknowledge the smearing of the boundary layers by applying a correction factor, to correctly interpret SPI camera images (Moser et al., 2021). Moreover, the lack of generic software and the absence of a well-established standard procedure limit the usefulness of SPI (Germano et al., 2011). This has been the case here with SpiArcBase software (Romero-Ramirez et al., 2013), from which we could not extract all parameters, as proper training of the software models (used for automatic measurements) was not achieved. Regarding data from the Van Veen grabs’ samples, the results on functional diversity indices should be interpreted with caution as the success of those analyses depends on the reliability of underlying data, knowledge on natural life-history of marine taxa and behaviour of species traits under different environmental conditions (Bremner, 2008). It would also be advisable to incorporate the use of the AZTI Marine Biotic Index (AMBI) or its multivariate version (M-AMBI) into the protocol (Borja et al., 2000; Borja et al., 2004). Indeed, those indices have proven to be highly efficient in the assessment of environmental changes induced by organic matter enrichment, such as it can occur in benthic ecosystems under mussel longlines aquaculture (Borja et al., 2009; Lacson et al., 2019; Marín et al., 2023). In underwater surveys employing video techniques, the majority rely on distance references such as lasers (Fields et al., 2019; Karatayev et al., 2018; Sheehan et al., 2010). This methodology simplifies density calculations compared to the current estimation. Furthermore, a combination of static and transect video surveys could benefit benthic characterisation (Tonk et al., 2019).

## Conclusions

Despite the individual limitations of the three monitoring techniques in this protocol, they have proven the usefulness of their joint use. Van Veen grabs enable a quantitative estimation of the biological and sedimentological data, offering an in-depth view on the benthic community composition (Van Hoey et al., 2014; Wilson et al., 2009). Macrofauna density, diversity and biomass can be used for structural community characterisation but also functional characterisation when linked with species traits datasets. Concurrently, SPI provides a detailed set of sedimentology and biogeochemical features, proving its cost, time-effectiveness and suitability when used as a complementary methodology (Grizzle & Penniman, 1991; Van Hoey et al., 2014). While SPI primarily addresses biogeochemistry in ecosystem functioning, trait-based approaches from grabs offer insights into additional aspects of functional diversity, including longevity, feeding, or development mode. Finally, diving video surveys have ensured seafloor characterisation, with detailed information about biogenic reef development.

In conclusion, the integration of Van Veen grabs, SPI and diving video surveys into the monitoring protocol of Coastbusters projects has allowed a holistic view of biodiversity and ecosystem functioning in the study area, providing a better understanding of environmental changes during the implementation of nature-based solutions. This study revealed that the assessed biological, biogeochemical and functional features extensively reflect the differences between the sheltered and exposed areas. Each technique brings a different, yet complementary, approach to the assessment of biological, physical and functional characteristics, allowing an integrated understanding of ecosystem processes (Birchenough et al., 2012; Van Hoey et al., 2014).

## Data Availability

The datasets generated during and/or analysed during the current study are available by contacting the project responsible (gert.vanhoey@ilvo.vlaanderen.be).
